# Effects of exercise dose based on the ACSM recommendations on depression in hemodialysis patients: a systematic review and meta-analysis of randomized controlled trials

**DOI:** 10.3389/fphys.2024.1513746

**Published:** 2025-01-31

**Authors:** Yang Fang, Bai Xiaoling, Li Huan, Guan Yaping, Zhang Binying, Wang Man, Wu Juan, Liu Xinyu

**Affiliations:** ^1^ Department of Nephrology, Guizhou Provincial People’s Hospital, Guiyang, Guizhou, China; ^2^ School of Nursing, Guizhou University of Traditional Chinese Medicine, Guiyang, Guizhou, China; ^3^ Department of Nursing, Guizhou Nursing Vocational College, Guiyang, Guizhou, China; ^4^ Hospital infection Management Department, Guizhou Provincial People’s Hospital, Guiyang, Guizhou, China; ^5^ School of Nursing, Zunyi Medical University, Zunyi, Guizhou, China; ^6^ Department of Nursing, The Affiliated Hospital of Guizhou Medical University, Guiyang, Guizhou, China

**Keywords:** hemodialysis, exercise, ACSM, depression, systematic review

## Abstract

**Objective:**

To explore the impact of various exercise doses on depressive symptoms among hemodialysis patients and offer valuable guidance for the selection of optimal exercise doses in clinical practice settings.

**Methods:**

A comprehensive systematic review was conducted across four major databases, namely, PubMed, Embase, Web of Science, and Cochrane Library, covering the period from their inception until August 2024. Exercise interventions were classified based on adherence to American College of Sports Medicine (ACSM) recommendations, dividing studies into groups with high and low/uncertain ACSM adherence. A meta-analysis was performed utilising Review Manager5.4.1 to assess the effects of ACSM adherence on depression in hemodialysis patients.

**Results:**

This meta-analysis incorporated a total of 19 randomized controlled trials, involving 1,285 patients. The mean age of the patients ranged from 33.2 to 70 years, and the average body mass index (BMI) fluctuated between 23.3 and 28.81 kg/m^2^. Males accounted for a relatively larger proportion of the participants. Among these trials, 14 were classified as having high ACSM adherence, while 5 were categorized as having low or uncertain adherence. Overall, exercise markedly improved depression in hemodialysis patients (SMD: −0.63, 95% CI: −0.87, −0.39; *p* < 0.05). The high ACSM adherence group showed greater improvement relative to the low/uncertain adherence group (SMD: −0.66 vs. −0.56). No notable disparities were noted in the effects of exercise duration or patient age on depression outcomes between the subgroups (*p* = 0.86, *p* = 0.48).

**Conclusion:**

Exercise interventions that exhibit high adherence to the ACSM guidelines prove to be more efficacious in alleviating depression among hemodialysis patients as compared to those with low or uncertain adherence levels.

**Systematic Review Registration:**

https://www.crd.york.ac.uk/prospero/#myprospero

## 1 Introduction

The latest global burden of kidney disease report reveals that the international incidence of chronic kidney disease (CKD) attained 9.5% in 2022, which has become one of the most significant burdens on the global public health system (Bello et al., 2024; Jha et al., 2023). The terminal phase of CKD, namely, end-stage renal disease (ESRD), exhibits a global prevalence ranging from 8% to 16%, and this prevalence is on an upward trajectory over time (Kramer et al., 2018). Hemodialysis (HD) is one of the principal treatments for patients with ESRD, and it can enhance their prognosis and quality of life. However, depression is one of the most prevalent complications among HD patients and can trigger increased mortality and hospitalisation rates (Iida et al., 2020; Alshelleh et al., 2023). Research has demonstrated that more than a quarter of HD patients is diagnosed of major depression (Kalra et al., 2024). A systematic review and meta-analysis involving 80,932 CKD patients from 27 countries indicated a 30.6% incidence rate of depression in HD patients (Adejumo et al., 2024). The study results revealed that the occurrence of depression might lead patients who had previously undergone HD to discontinue dialysis (Silva et al., 2022; Virani et al., 2021), and treatment adherence would decrease (Cogley et al., 2022). Simultaneously, the probability of losing severe function would increase by 46% (Virani et al., 2021). Generally speaking, the incidence rate of depression among HD patients is relatively high, and the occurrence of depression will have numerous adverse effects on the health of HD patients. Therefore, the early and efficient management of depressive symptoms in HD patients are of paramount importance.

Current evidence supporting the role of exercise in mental health among clinical populations appears to be popular in the exercise community (Newsome et al., 2024). The treatment modalities for depression in HD patients incorporate both pharmacological and non-pharmacological approaches (Kubanek et al., 2021; Grigoriou et al., 2015; Nadort et al., 2020). Prolonged use of antidepressants in patients with CKD may exacerbate renal impairment, lead to adverse drug reactions and lead to poor treatment adherence (Menezes et al., 2021). Therefore, exercise, as a non-drug treatment approach,has become an essential means to improve the psychological and physiological wellbeing of the HD population (Gerogianni et al., 2019; Sovatzidis et al., 2020). Numerous studies indicate that exercise significantly relieves fatigue among HD patients (Lu et al., 2024) and promotes cardiovascular health (Davies et al., 2023), as well as ameliorates sarcopenia and physical function. Physical inactivity among HD patients also serves as a robust predictor of mortality (Huang et al., 2019). Moreover, exercise has been demonstrated to markedly alleviate depression symptoms and improve the overall wellbeing of individuals with HD (Greenwood et al., 2021; Yu et al., 2023; Bernier-Jean et al., 2022; Hargrove et al., 2021). The Centers for Disease Control and Prevention (CDC) advocates that physical activity serves as a preventive against conditions like depression, anxiety, cognitive decline and dementia (Halle et al., 2024). The American Kidney Foundation also underscores the importance of exercise in managing complications in HD patients (Huang et al., 2019). The 2021 guidelines for CKD by the British Society of Nephrology suggest that HD patients without contraindications should engage in at least 30 min of exercise 3–5 times weekly (Baker et al., 2022; Lambert et al., 2022). The effect of exercise seems to be dose-dependent. A recent consensus statement document from the Italian Society of Nephrology highlights that physical activity and exercise prescriptions should be tailored to each patient, considering factors such as physical function, comorbidities, space availability, and time to ensure adequacy, safety, and feasibility (Battaglia et al., 2024). In summary, *l*th/fitness-related outcomes in this cohort should be highlighted, focusing on the popularity. Newsome et al. (2024), safety, and effectiveness (Sovatzidis et al., 2020; Yu et al., 2023; Hargrove et al., 2021) of this particular exercise modality among people with or without health issues. However, there are few studies on exercise dose for depression in HD patients, and standardising exercise interventions remains a challenge. Therefore, further studies are required to investigate the optimal exercise dose for HD patients.

In 2014, the American College of Sports Medicine (ACSM) issued guidelines for the prescription exercise, emphasizing aerobic exercise, resistance training and flexibility exercises (Ferguson, 2014). Nevertheless, it remains unclear whether exercise programs that adhere to ACSM recommendations have a more significant influence on depression in HD patients. This systematic review seeks to evaluate the comparative outcomes of exercise interventions with high and low/uncertain adherence to ACSM suggestions on depression in HD patients.

## 2 Materials and methods

This systematic review and meta-analysis follows the Preferred Reporting Items for Systematic Reviews and Meta-Analyses (PRISMA; Page et al., 2021) guidelines and has been registered with PROSPERO (CRD42024579620).

### 2.1 Search strategy

A comprehensive systematic review was conducted across four major databases, namely, PubMed, Embase, Web of Science and Cochrane Library, covering the period from their inception until August 2024. Additionally, manual searches were performed for relevant studies not retrieved from the databases. If needed, the corresponding authors were contacted for further information.The detailed search strategy is detailed in Supplementary Appendix S1.

### 2.2 Eligibility criteria

Eligibility criteria were established using the PICOS framework: (1) Participants: patients aged 18 years or older undergoing HD as renal replacement therapy, excluding those who received HD due to acute renal failure. (2) Interventions:any form of exercise, including aerobic exercise, resistance training, flexibility exercise, or exercise combined with video technology. (3) Comparisons: the control group either received no treatment or a treatment unrelated to exercise. (4) Outcomes:the primary endpoint was depression, assessed using tools such as the Beck Depression Inventory, The Center for Epidemiological Scale-Depression, or Zung Self-Rating Depression Scale. The Beck Depression Inventory is mainly used in clinical assessment and research to accurately assess the severity of depression. The Center for Epidemiological Scale-Depression focuses on large-scale epidemiological studies that screen populations for depressive symptoms. Zung Self-Rating Depression Scale is used for preliminary clinical judgment and mental health investigation, and also has a certain degree of severity. (5) Study design: randomised controlled trial (RCT).

The following studies were excluded: (1) Animal studies. (2) Reviews, case reports and conference abstracts. (3) Studies with missing data that could not be obtained by contacting the authors. (4) Duplicate publications, or studies without full-text access. (5) Case-control studies, cross-sectional studies, and longitudinal studies were excluded.

### 2.3 Data synthesis and analysis

Data were autonomously screened by two researchers (FY and HL) according to the established inclusion and exclusion criteria. When discrepancies arose, a third investigator (XB) adjudicated. Relevant data were extracted and recorded in Excel, encompassing the title, first author, year of publication, country, sample size, intervention details, age, intervention frequency, exercise intensity, duration and type of exercise. For investigations with several follow-up points, only the data immediately post-intervention were gathered. When multiple assessment tools were used for the same outcome, the most appropriate tool was selected based on a predetermined hierarchy.

Following data extraction, both authors independently assessed the exercise intervention’s dosage (encompassing frequency, intensity, workload and duration) and compliance in HD patients, as per ACSM guidelines (Table 1) (Garber et al., 2011).

**TABLE 1:** The ACSM’s prescriptions for cardiorespiratory fitness, muscular strength, and flexibility in adults seemingly in good health.

Exercise dose	Cardiorespiratory exercise	Resistance exercise	Flexibility exercise
Frequency	3–5 days/week	2–3 days/week	≥2–3 day/week, daily
Intensity/workload	40%–60% VO2R or HRR of 12–13 on a 6–20 scale	Start with 40%–50% 1RM, more capable with 60%–70% 1RM	Stretch until you feel your muscles being pulled tight or a slight discomfort
Duration	Continuous or cumulative 30 min	Starting with one set of 8–12 repetitions, increase to two sets after about 2 weeks. Perform no more than 8–10 exercises per session	Static stretching held for 10–30 s, repeated 2–4 times

Note: HRR, Heart rate reserve. VO2R, oxygen uptake reserve.

### 2.4 Statistical analyses

Each physical activity metric was evaluated using a 0–2 point scale: 2 points were awarded for fulfilling the requirements, 1 point for ambiguity, and 0 points for failing to meet the criteria. When discrepancies arose in the evaluation process, the researchers consulted with an additional author to achieve agreement. Utilising this assessment method, the percentage of exercise regimens conforming to ACSM-suggested guidelines was determined for every investigation. A percentage of ≥75% signified strong compliance with ACSM recommendations, whereas <75% indicated low or questionable adherence.

Meta-analysis was executed utilising Review Manager 5.4.1 software. For data characterised by non-normal distribution and reported as medians (M) with interquartile ranges (P_25_, P_75_) in the original studies, the methods described by McGrath et al. (2020) were employed to calculate the mean value ±standard deviation (SD). The standardised mean difference (SMD) was used as the effect measure, and the Higgins I^2^ statistic assessed statistical variability across investigations. A fixed-effect model was deemed appropriate when treatment effects were homogeneous and heterogeneity was low (*I* ^*2*^ < 50%). Conversely, a random-effects model was employed when heterogeneity was significant (*I* ^*2*^ > 50%), with effect sizes denoted as SMD and 95% confidence intervals (95%CI). The investigations were categorised into two groups grounded in adherence to ACSM recommendations: high adherence and low or uncertain adherence. Publication bias was elevated by generating a funnel plot to evaluate the effect size against the standard deviation for each investigation, with a significance threshold established at *p* < 0.05. Sensitivity analysis was meticulously carried out by systematically omitting each of the included studies one by one with the application of Stata 17.0.

### 2.5 Quality appraisal

The methodological rigour of the incorporated investigations was autonomously evaluated by two separate teams of researchers employing the Cochrane (Higgins et al., 2011) risk of a bias assessment tool for RCTs. This appraisal encompassed six domains: random sequence generation (selection bias), allocation concealment (selection bias), participant and personnel blinding (performance bias), outcome data (attrition bias), reporting bias and other biases. The risk of bias was categorised into three categories: low risk, high risk and unclear risk.

## 3 Results

### 3.1 Study selection

**FIGURE 1:**
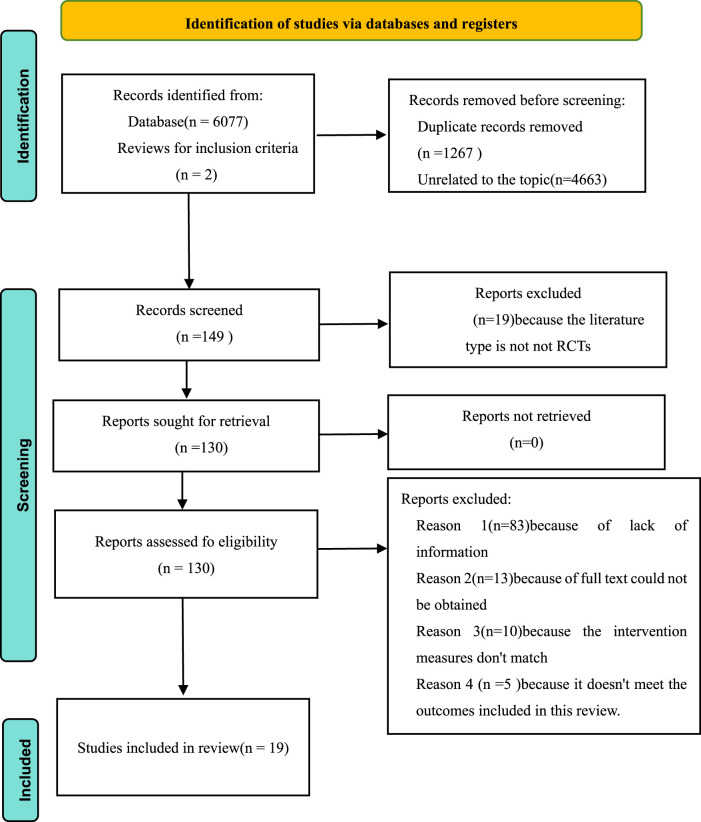
PRISMA Study flow diagram.

Altogether 6,077 publications were procured from four databases (PubMed: 2,065; Embase: 1,853; Web of Science: 1,027; Cochrane Library: 1,132), with 2 additional documents manually identified from alternative sources. After examining titles and abstracts, 149 articles were chosen for comprehensive evaluation. Ultimately, 19 investigations fulfilled the eligibility requirements and were incorporated into this analysis (Carney et al., 1987; Kouidi et al., 1997; van Vilsteren et al., 2005; Cheema et al., 2007; Sakkas et al., 2008; Ouzouni et al., 2009; Kouidi et al., 2010; Giannaki et al., 2013; Liu et al., 2015; Dziubek et al., 2016; Tang et al., 2017; Rahimimoghadam et al., 2017; Ortega-Pérez et al., 2020; Lin et al., 2021; Deus et al., 2021; Liu et al., 2023; Maynard et al., 2019; Valenzuela et al., 2018; Walklin et al., 2023) (Figure 1).

### 3.2 Study characteristics

Table 2 depicts an overview of the primary attributes of the incorporated studies. In total, 19 investigations involved 1,285 participants, ranging in age from 33.2 to 70 years and the average body mass index (BMI) fluctuated between 23.3 and 28.81 kg/m^2^. Males accounted for a relatively larger proportion of the participants. The experimental groups included 667 participants, while the control groups comprised 618 participants. Intervention periods varied from 8 weeks to 12 months. Nine investigations focused on aerobic exercise, four on resistance exercise, one on flexibility exercises, four on a blend of aerobic and resistance exercises and one on a blend of aerobic and flexibility exercises. The depressive function was assessed using the Beck Depression Inventory (BDI) in nine studies, the Zung Self-Rating Depression Scale in three studies and Center for Epidemiological Scale-Depression (CES-D) in two studies. Additional tools used in a few studies included the Geriatric Depression Scale, the Hospital Anxiety and Depression Scale (HADS), Self-Rating Depression Scale (SDS), the General Health Questionnaire-28 (GHQ-28) and the Patient Health Questionnaire-4 (PHQ-4). Geographically, the studies were conducted in Greece (5), China (4), Spain (2), Brazil (2), New Zealand (2), and one study each in the United Kingdom, the Netherlands, Poland, Iran and the United States (Table 2).

**TABLE 2:** Fundamental characteristics of the study.

Author, year	Country	Age mean (SD)	BMI (kg/m^2^) mean (SD)	Total/male/female	Interventions	Length of intervention	Measuring tools	Outcome mean (SD)
Carney et al. (1987)	Unites States	T:36.1 (3.2) C:40.7 (5.3)	NR	T:10/5/5 C:7/3/4	Aerobic exercise	6 months	BDI	T:7.7 (1.9) C:7.2 (1.3)
Kouidi et al. (1997)	Greece	T:49.6 (12.1) C:52.8 (10.2)	NR	T:20/11/9 C:11/4/7	Aerobic exercise	6 months	BDI	T:13.7 (9.5) C:21.3 (11.9)
van Vilsteren et al. (2005)	Netherlands	T:52 (15) C:58 (16)	NR	T:53/34/19 C:43/30/13	Aerobic exercise	12 weeks	Zung	T:37.2 (8.3) C:41.4 (9.6)
Cheema et al. (2007)	New Zealand	T:60.0 (15.3) C:65.0 (12.9)	T:27.0 (6.0) C:28.0 (5.7)	T:24/17/7 C:25/17/8	Resistive exercise	12 weeks	GDS	T: 0.3 (3.6) C:1.0 (2.9)
Sakkas et al. (2008)	Greece	T:48 (14) C:70 (11)	T:26.0 (5.0) C:27.0 (7.0)	T:7/5/2 C:7/5/2	Aerobic and resistance exercises	16 weeks	Zung	T:38 (11) C:46 (5)
Ouzouni et al. (2009)	Greece	T:47.4 (15.7) C:50.5 (11.7)	NR	T:19/14/5 C:14/13/1	Aerobic and Flexibility exercise	10 months	BDI	T:11.7 (3.6) C:19.4 (4.0)
Kouidi et al. (2010)	Greece	T:46.3 (11.2) C:45.8 (10.9)	NR	T:24/14/10 C:20/12/8	Aerobic exercise	12 months	BDI; HADS	T:14.61 (4.15) C:22.10 (6.24)
Giannaki et al. (2013)	Greece	T:56.4 (12.5) C:56.8 (16.5)	T:27.0 (3.6) C:25.3 (1.7)	T:15/11/4 C:7/5/2	Aerobic exercise	6 months	Zung	T:35.84 (6.38) C:43.71 (11.17)
Liu et al. (2015)	China	T:44.3 (6.7) C:33.2 (7.0)	NR	T:10/6/4 C:10/5/5	Aerobic exercise	12 weeks	BDI-II	T:18.3 (9.9) C:30.6 (10.7)
Dziubek et al. (2016)	Poland	T:66.3 (13.1) C:56.4 (13.6)	NR	T:20/9/11 C:8/5/3	Aerobic and resistance exercises	6 months	BDI	T:11.9 (10.5) C:11.0 (6.3)
Tang et al. (2017)	China	T:46.26 (15.6) C:43.90 (12.4)	T:23.82 (3.76) C:23.30 (3.18)	T:42/28/14 C:42/23/19	Aerobic exercise	12 weeks	HAD-D	T:4.52 (2.62) C:6.40 (2.84)
Rahimimoghadam et al. (2017)	Iran	T:39.1 (2.2) C:38.4 (1.8)	NR	T:25/21/4 C:25/20/5	Flexibility exercise	8 weeks	GHQ-28	T: 8.6 (3.06) C:10.4 (2.4)
Ortega-Pérez et al. (2020)	Spain	T:62.2 (15.0) C:59.3 (16.1)	T:26.6 (3.7) C:25.1 (5.3)	T:24/15/C:22/14/8	Aerobic and resistance training	16 weeks	CES-D	T: 9.2 (8.7) C:14.5 (8.1)
Lin et al. (2021)	China	T:62.0 (9.5) C:62.1 (12.3)	T:23.4 (3.7) C:23.4 (4.5)	T:32/22/10 C:32/19/13	Aerobic exercise	12 weeks	BDI	T:5.0 (6.8) C:12.5 (9.2)
Deus et al. (2021)	Brazil	T:67.27 (3.24) C:66.33 (3.88)	T:27.30 (3.77) C:26.82 (2.90)	T:81/46/35 C:76/40/36	Resistive exercise	6 months	BDI	T:26.33 (6.28) C:27.28 (4.35)
Liu et al. (2023)	China	T:56.3 (11.10) C:59.2 (10.41)	NR	T:42/15/27 C:42/26/16	Aerobic exercise	24 weeks	SDS	T:30.39 (10.41) C:38.65 (9.20)
Maynard et al. (2019)	Brazil	T:49 (15.2) C:43.9 (11.7)	T:25.5 (5) C:24.5 (4.5)	T:20/12/8 C:20/10/10	Endurance and strength training combined with virtual reality	12 weeks	CES-D	T:7.1 (7.3) C:13.1 (9.4)
Valenzuela et al. (2018)	Spain	T:68 (13) C:68 (11)	T:27 (6) C:27 (5)	T:27/18/9 C:40/29/11	Endurance-resistance training program	14 weeks	BDI	T: 8 (7) C:14 (10)
Walklin et al. (2023)	United Kingdom	T:53.9 (13.6) C:53.8 (13.5)	T:27.98 (1.62) C:28.81 (1.51)	T:173/96/77 C:167/89/78	Kidney BEAM:Aerobic and resistance training	14 weeks	PHQ-4	T:2.35 (3.74) C:2 (2.99)

Note: SD: standard deviation, NR:not report, T: experimental group, C: control group, BDI: Beck Depression Inventory; SDS:Self-rating Depression Scale; GDS:Geriatric Depression Scale; Zung:Zung self-rating Depression Scale; HADS: hospital anxiety and depressive depression scale; HAD-D:depression subscale; GHQ-28:general health questionnaire-28; CES-D:The Center for Epidemiological Scale-Depression; PHQ-4:The Patient-Health Questionnaire-4.

### 3.3 Risk of bias

**FIGURE 2:**
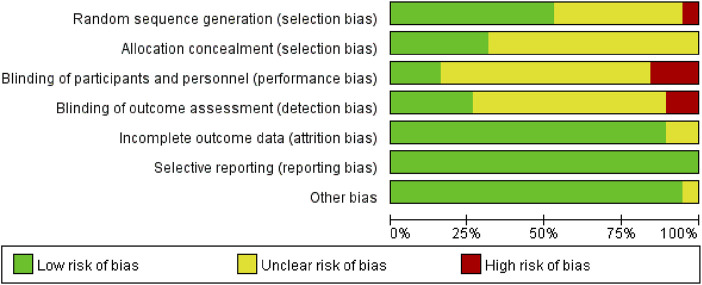
The combined risk of bias percentage in each risk domain for all included studies.

**FIGURE 3:**
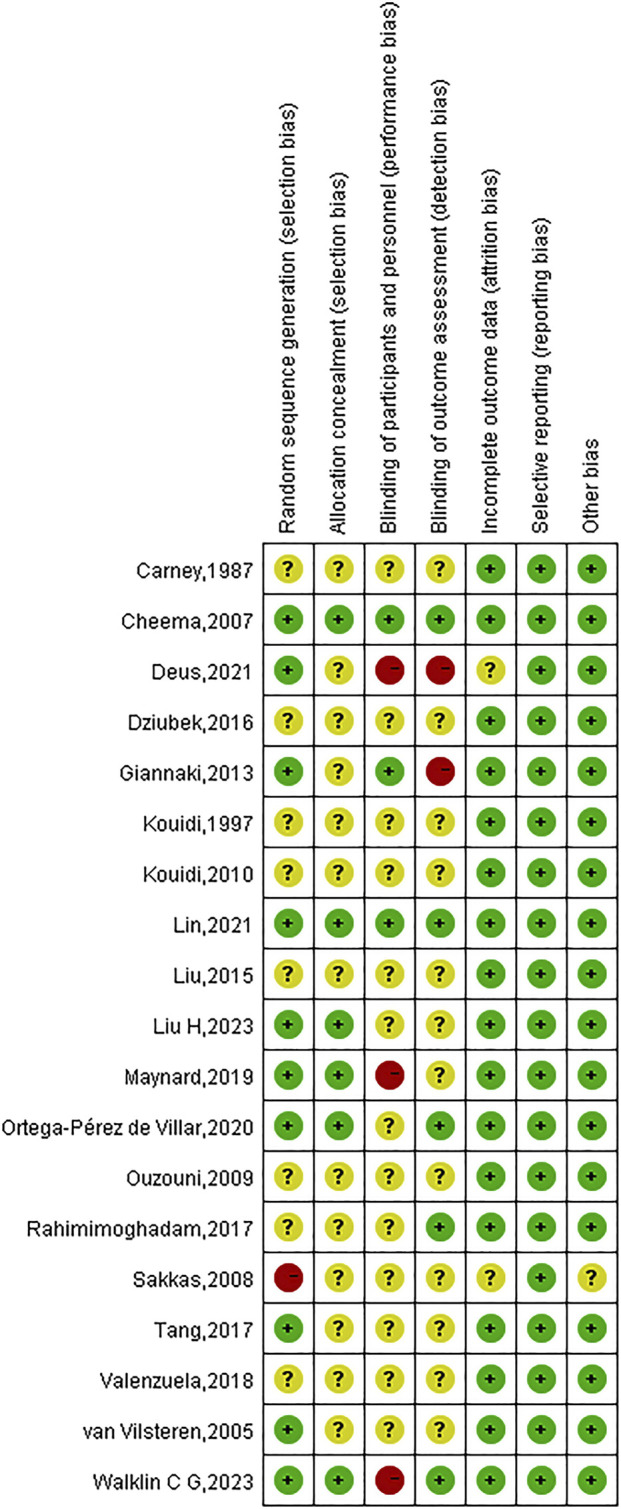
Summarized risk of bias for all exercise studies.

Due to incomplete methodological descriptions, the risk of bias in the examined research was largely categorised as high or uncertain. All 19 studies reported using randomisation, but only 10 (52.63%) provided detailed information on random sequence generation, and 6 studies described appropriate allocation concealment. Given the inherent challenges in blinding both participants and investigators in exercise interventions, only three studies blinded participants and five studies used blinded outcome assessments. Attrition rates were reported in most trials (89.47%), and complete data were available for all 19 studies. An in-depth evaluation of the calibre of the incorporated studies is depicted in Figures 2, 3.

### 3.4 Adherence to the ACSM guidelines

Following ACSM guidelines, the experimental groups were divided into two categories: high adherence and low or uncertain adherence. In 14 studies, physical activity achieved a compliance rate of ≥75%, meeting ACSM recommendations. In contrast, five studies had adherence rates of <75%, primarily due to experimental designs that did not incorporate all the recommended parameters. Additionally, a lack of sufficient detail on exercise prescription hindered accurate assessment of adherence (Table 3).

**TABLE 3:** Evaluation of compliance with ACSM.

Author, year	Cardiorespiratory exercise			Resistance exercise				Flexibility exercise			ACSM consistency
	Frequency	Intensit/Workload	Duration	Frequency	Intensity/Workload	Repetitions	Sets	Frequency	Intensity/Workload	Duration	Points (Percent%)
	3–5 day/week	40%–60% VO2R or HRR; 64%–76% HRmax; RPE of 12–13 on a 6–20 scale	Continuous or cumulative 30 min	2–3 days/week	Start with 40%–50% 1RM, more capable with 60%–70% 1RM	8–12	≥1group	≥2–3 day/week, daily	Stretch until you feel your muscles being pulled tight or a slight discomfort	Static stretching held for 10–30 s, repeated 2–4 times	
Carney et al. (1987)	3 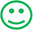	VO2R:60%–65% 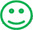	45–60 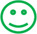								6/6 (100)
Kouidi et al. (1997)	3–4 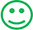	VO2R:50%–60%; HRmax:60%–70% 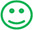	90 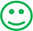								6/6 (100)
van Vilsteren et al. (2005)	2–3 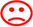	HRmax:60% 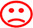	30 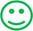								2/6 (33.33)
Cheema et al. (2007)				3 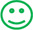	Ind.tail 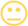	8 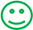	2 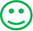				7/8 (87.5)
Sakkas et al. (2008)	3 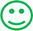	45–50 rpm 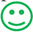	45 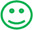	3 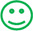	RM:65–75 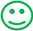	NR 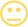	NR 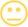				12/14 (85.71)
Ouzouni et al. (2009)	3 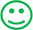	HRR:13–14 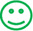	30 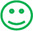					3 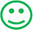	NR 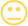	NR 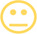	10/12 (83.33)
Kouidi et al. (2010)	3 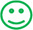	VO2R:70%; 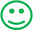 RPE:11–13	60–90 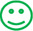								6/6 (100)
Giannaki et al. (2013)	3 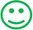	HRmax:60%–65% 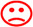	NR 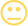								3/6 (50)
Liu et al. (2015)	3 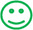	RPE:11–13 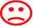	30 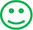								4/6 (66.67)
Dziubek et al. (2016)	3 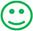	RPE:5–6 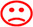	35–50 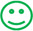	3 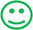	Ind.tail 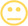	30 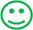	4–5 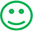				11/14 (78.57)
Tang et al. (2017)	3 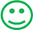	RPE:12–15 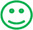	30 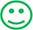					3 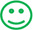	NR 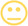	NR 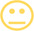	10/12 (83.33)
Rahimimoghadam et al. (2017)								3 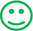	NR 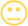	4 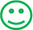	5/6 (83.33)
Ortega-Pérez et al. (2020)	3 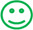	RPE:12–15 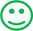	10–30 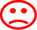	3 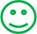	NR 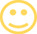	10 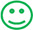	1–3 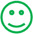				11/14 (78.57)
Lin et al. (2021)	3 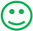	RPE:12–14 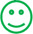	30 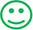								6/6 (100)
Deus et al. (2021)				3 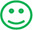	NR 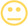	12 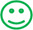	3 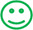				7/8 (87.5)
Liu et al. (2023)	3 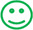	VO2 peak:50% 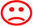	30 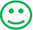								4/6 (66.67)
Maynard et al. (2019)	3 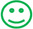	Ind.tail 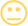	30–60 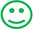								5/6 (83.33)
Valenzuela et al. (2018)	3 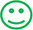	scale:12–14 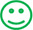	30 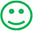	3 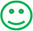	NR 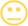	NR 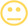	NR 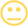				11/14 (78.57)
Walklin et al. (2023)	NR 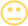	NR 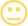	20–30 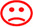	2 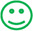	NR 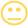	NR 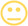	NR 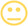				7/14 (50)

Note: ACSM, American College of Sports Medicine. Ind. tail, individually tailored. NR, not reported. Happy/green face, fulfils recommendation (2 points), neutral/yellow face, uncertain fulfilment (1 point), unhappy/red face, does not fulfil recommendation (0 points).

### 3.5 Meta-analysis

**FIGURE 4:**
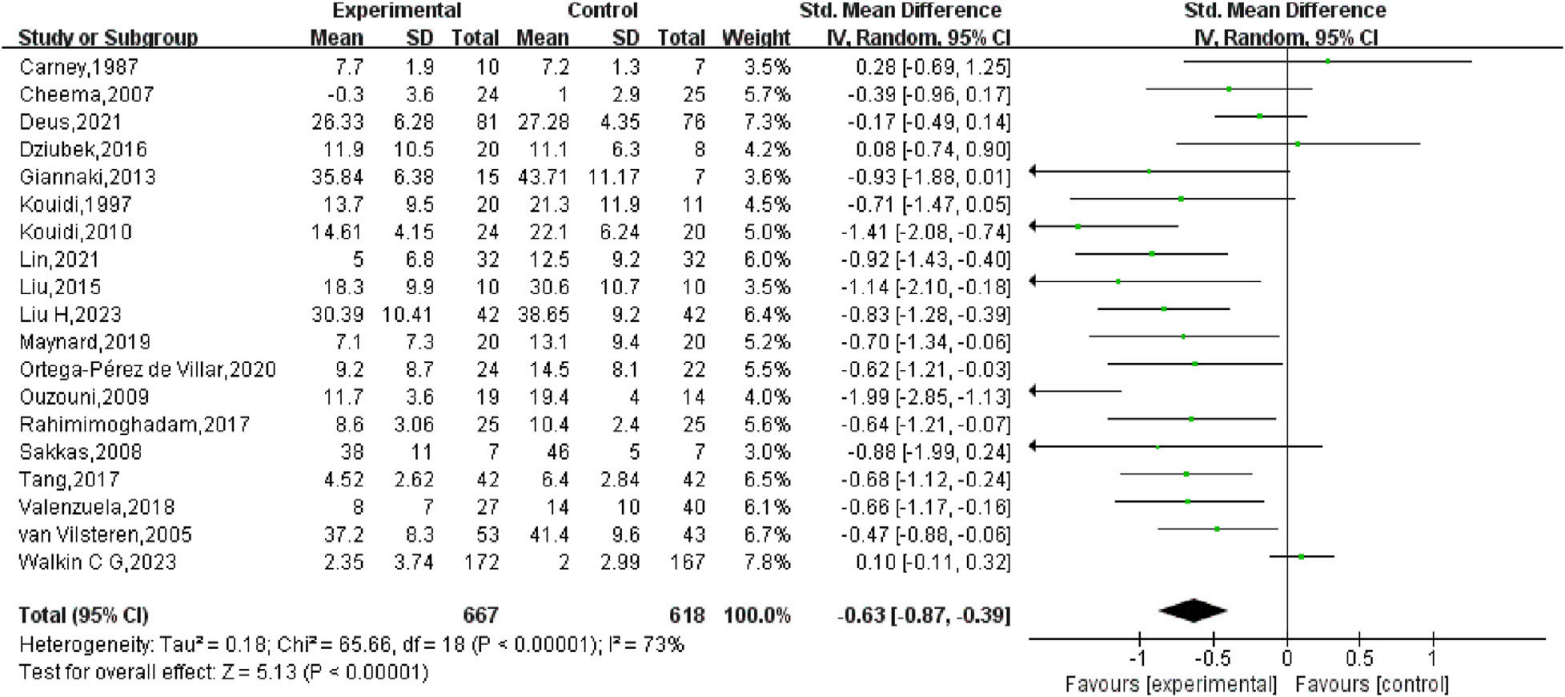
Forest plot of the impact of exercise on depression in hemodialysis patients.

First, a comprehensive heterogeneity assessment of the incorporated studies was conducted, indicating substantial heterogeneity (*I* ^*2*^ = 73%, *p* < 0.01). Consequently, a random-effects model was employed for statistical analysis. The outcomes suggested that exercise interventions in the experimental group had a greater positive effect on depression relative to the control group. The overall SMD was −0.63 (95% CI: −0.87, −0.39), with statistically significant results (*p* < 0.01), indicating that exercise exerted a considerable effect in alleviating depression in HD patients (Figure 4).

**FIGURE 5:**
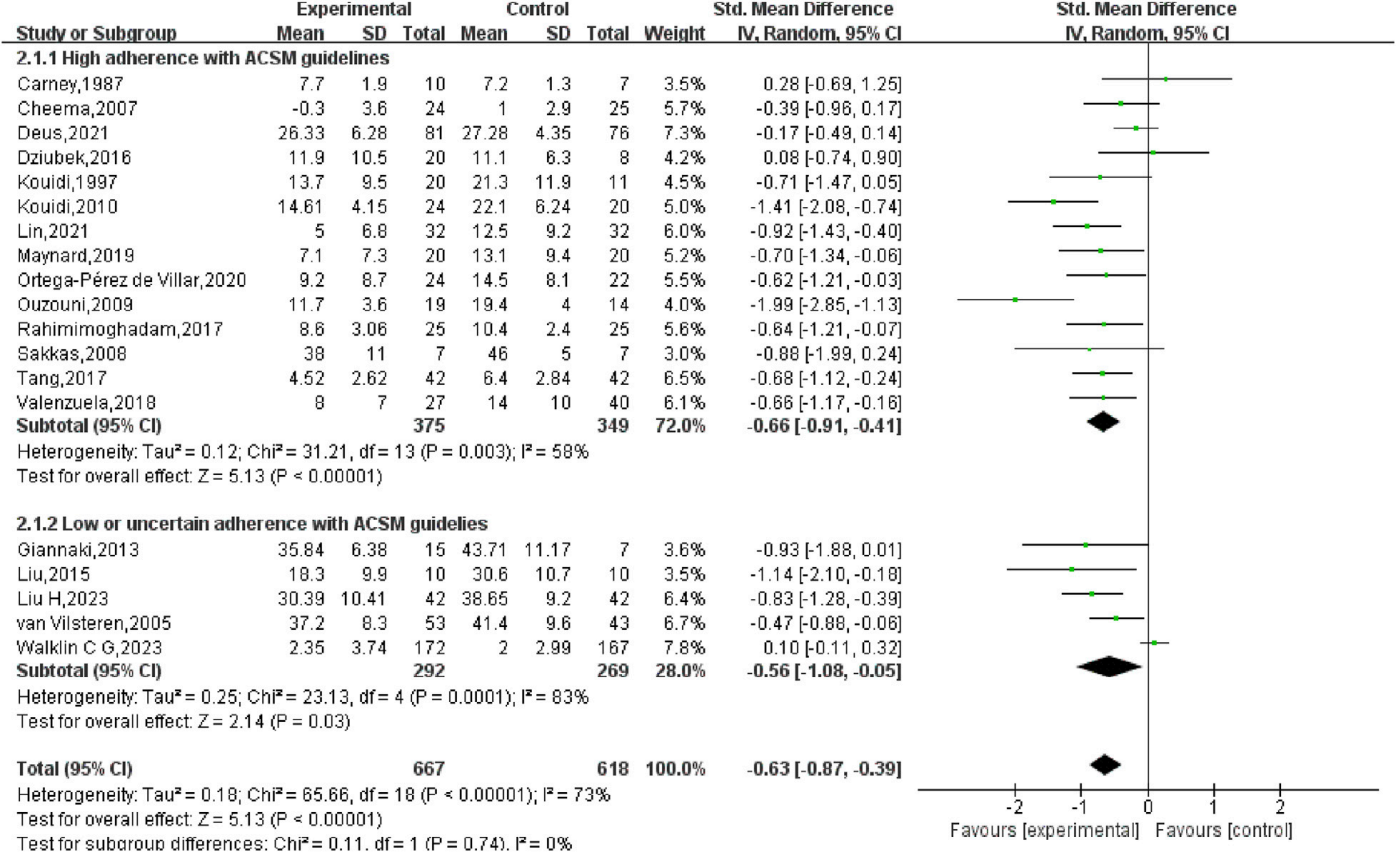
Subgroup analysis of the influence of exercise on depression among hemodialysis patients.

Subgroup analysis indicated that in the high adherence group, the SMD was −0.66 (95% CI: −0.91, −0.41), with moderate heterogeneity (*I* ^*2*^ = 58%) and a statistically notable difference (*p* < 0.01). This suggests that exercise prescriptions closely aligned with ACSM recommendations had a considerable effect on reducing depression in HD patients. In the low or uncertain adherence group, the SMD was −0.56 (95% CI: −1.08, −0.05), with high heterogeneity (*I* ^*2*^ = 83%) and a statistically notable difference (*p* = 0.03). This indicates that even exercise interventions with lower or uncertain adherence to ACSM guidelines had a significant effect on depression in HD patients. When comparing the two groups, exercise interventions highly adherent to ACSM guidelines demonstrated a slightly stronger effect on depression reduction (SMD: high adherence −0.66 vs low/uncertain adherence −0.56) (*p* < 0.05) (Figure 5).

**FIGURE 6:**
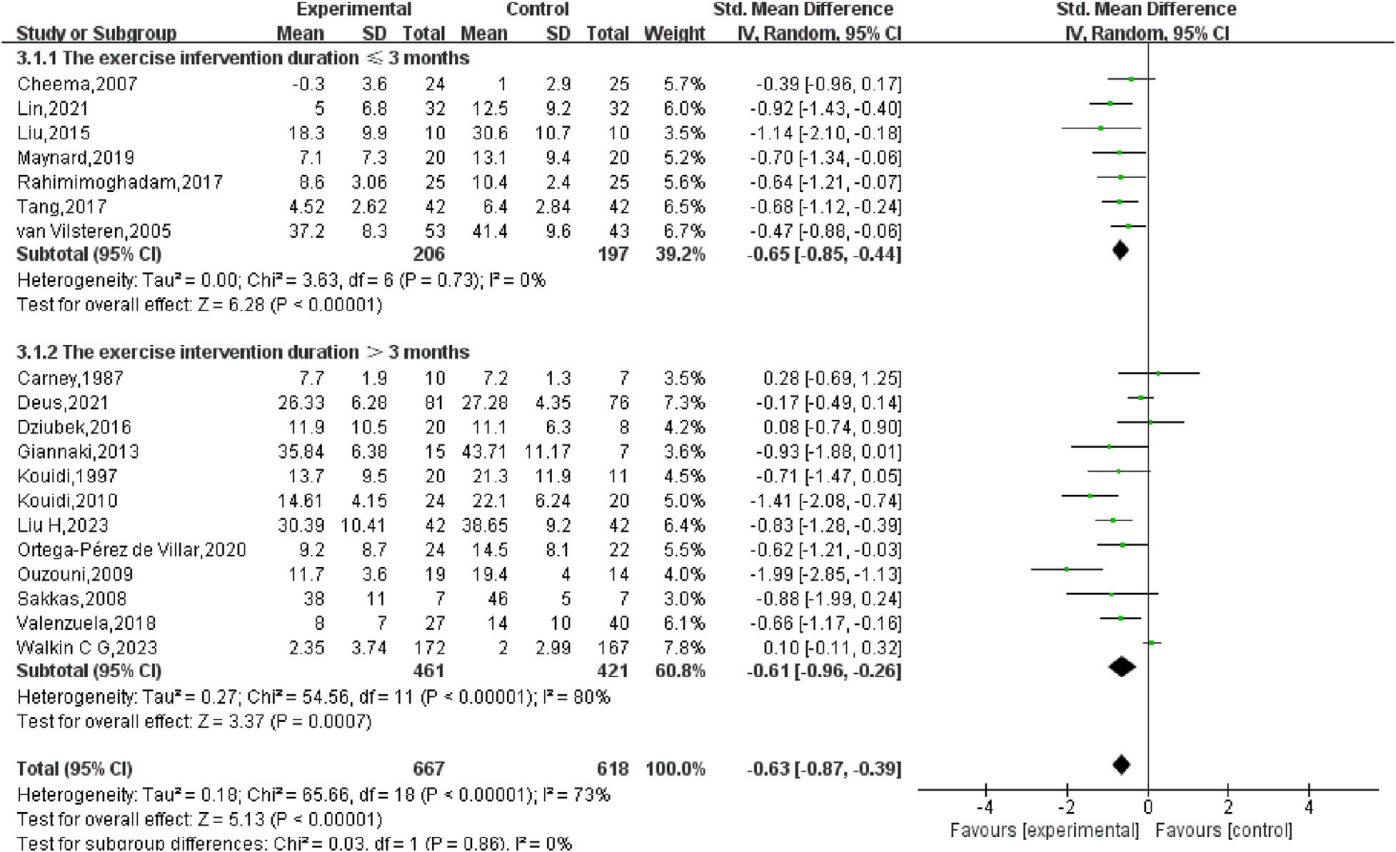
Subgroup analysis of the intervention duration for depression in hemodialysis patients.

A subgroup analysis grounded in the length of the exercise intervention (ranging from 8 weeks to 12 months) was also performed. The results showed that for interventions lasting ≤3 months, the SMD was −0.65 (95% CI: −0.85, −0.44) (*p* < 0.01), while for interventions lasting >3 months, the SMD was −0.61 (95% CI: −0.96, −0.26) (*p* < 0.01). The comparison between the two groups yielded no statistically significant disparity (*p* = 0.86), suggesting that the intervention’s length did not markedly affect the overall results (Figure 6).

**FIGURE 7:**
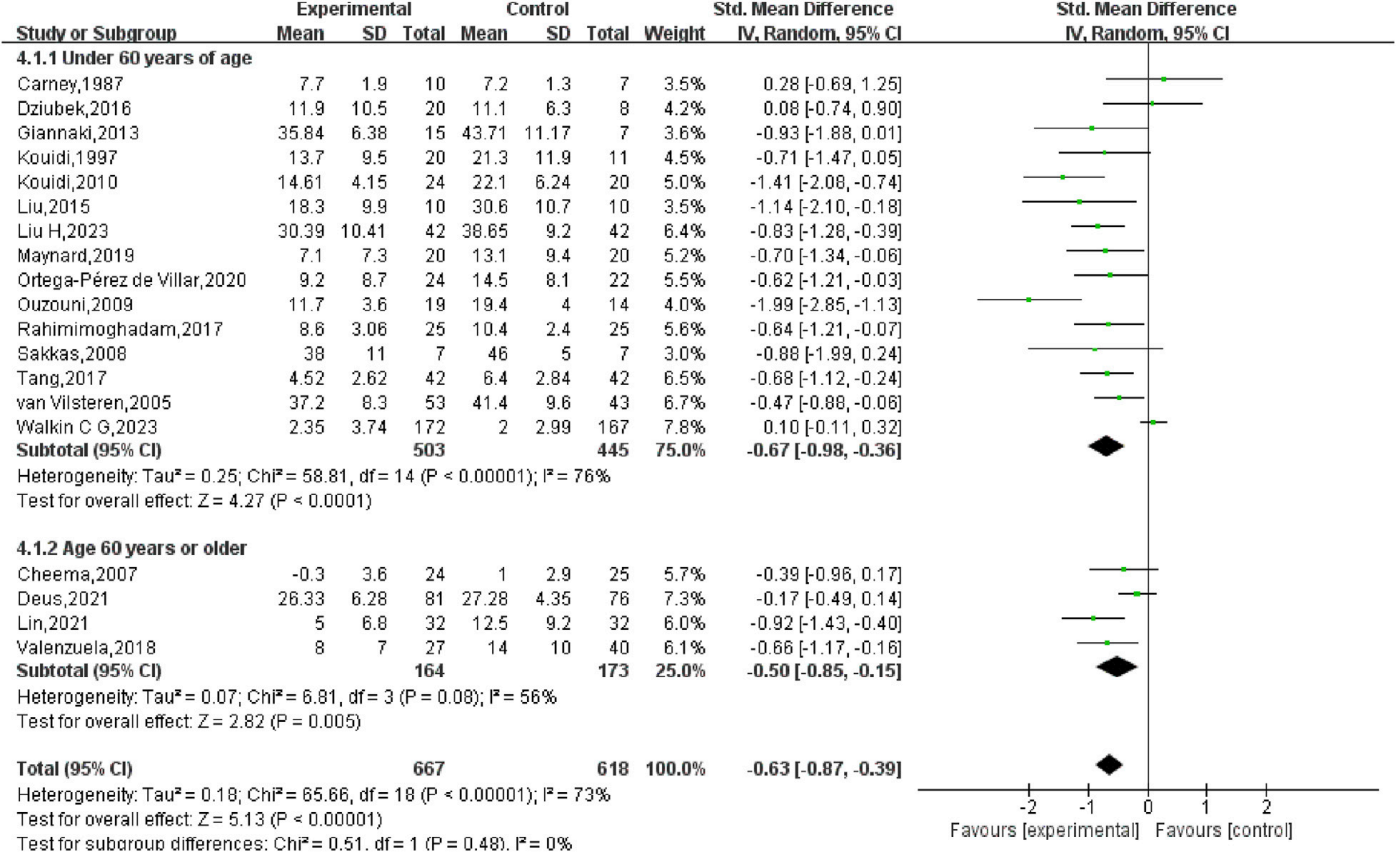
Subgroup analysis of the age factor in depression among hemodialysis patients.

Given the wide age range of participants (33.2–70 years), a subgroup analysis based on age was also conducted. For participants younger than 60 years, the SMD was −0.67 (95% CI: −0.98, −0.36) (*p* < 0.01), while for participants aged 60 years or older, the SMD was −0.50 (95% CI: −0.85, −0.15) (*p* < 0.01). The analysis revealed no statistically significant disparity between the two age cohorts (*p* = 0.48), indicating that age did not markedly influence the overall results (Figure 7).

**FIGURE 8:**
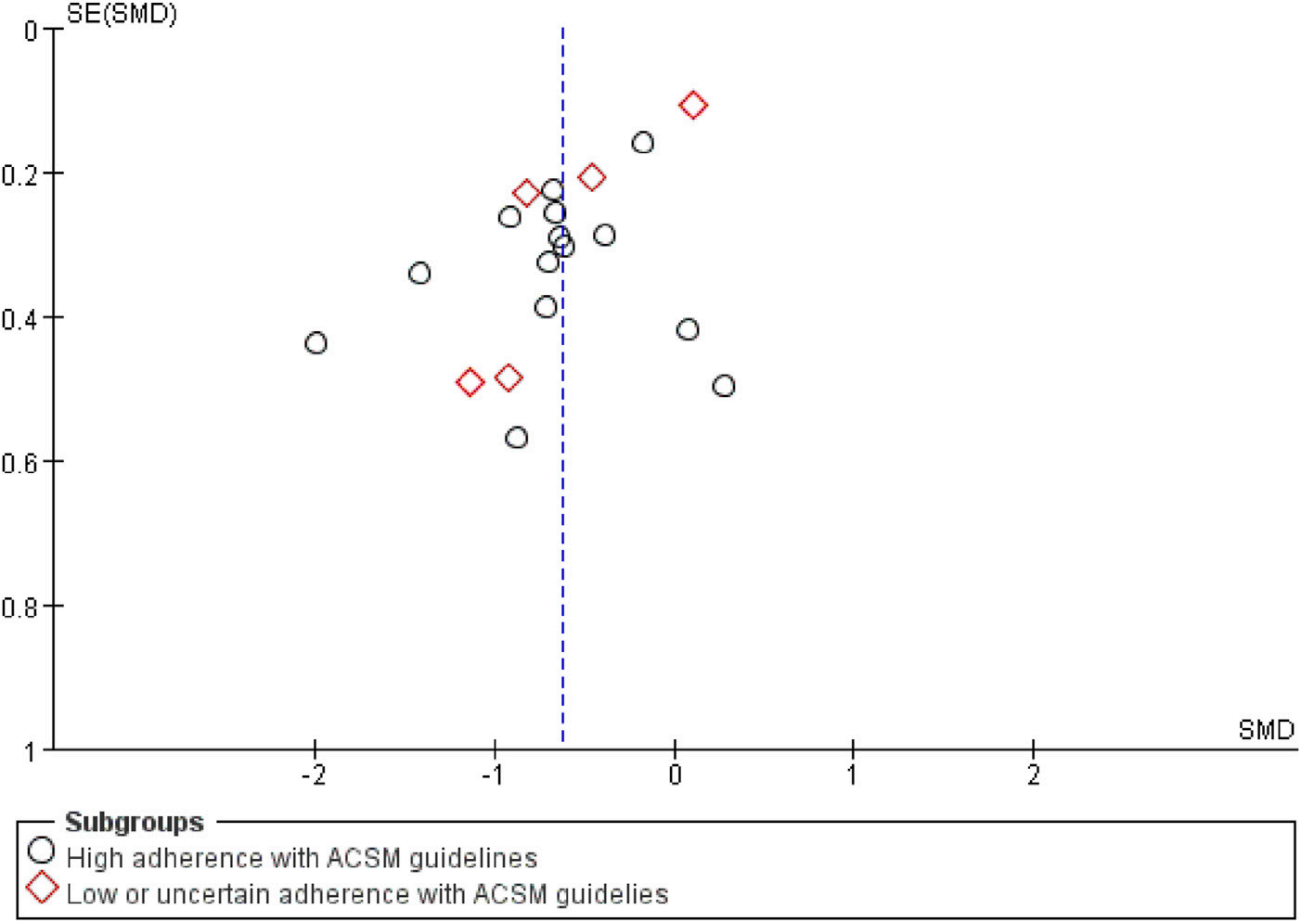
Funnel plots were incorporated in the study.

**FIGURE 9:**
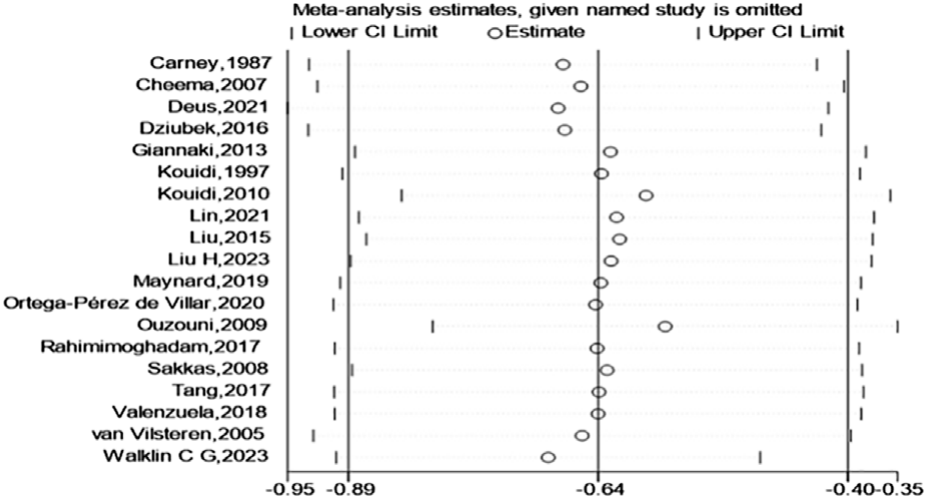
Sensitivity was incorporated in the study.

Finally, a test for publication bias was carried out by means of a funnel plot (Figure 8). The distribution of studies on both sides of the funnel plot seemed fairly balanced, implying the absence of significant publication bias. According to the sensitivity analysis (Figure 9), we found that no single study had a significant impact on the overall results, indicating the robustness of our research findings.

## 4 Discussion

This investigation compared the effects of exercise prescriptions with high adherence to the ACSM recommendations against those with low or uncertain adherence on depression in HD patients. The analysis encompassed 19 investigations involving 1,285 subjects.

The results confirmed that exercise remains a highly effective non-pharmacological intervention for alleviating depressive symptoms in HD patients (SMD: −0.63, 95% CI: −0.87, −0.39, *p* < 0.05), consistent with previous findings (Yu et al., 2023; Chung et al., 2017; Rezaei et al., 2015; Song et al., 2018; Hargrove et al., 2021; Wen et al., 2020). The mechanisms through which exercise reduces depressive symptoms are diverse and include the regulation of serotonin and norepinephrine levels, brain-derived neurotrophic factor and various immune-inflammatory pathways (Ross et al., 2023). However, prior investigations have emphasised the effectiveness of different exercise interventions varies. Consequently, investigating the impact of exercise doses on depressive symptoms in this patient population. Meta-analyses by Yu et al. (2023) and Hargrove et al. (2021) indicated that aerobic exercise is more effective than combined or resistance exercise in reducing depression levels. Chung et al. (2017) and Rezaei et al. (2015) found that exercise during dialysis markedly improved depressive symptoms when compared to no exercise, no resistance exercise, or walking at home. Similarly, Gomes et al. showed that a combination of aerobic and resistance exercises markedly improved depressive symptoms in HD patients. Gomes et al. (2018) the intensity and frequency of exercise are also critical factors. Current evidence suggests that any exercise intervention lasting 2–12 months may improve depressive symptoms, with sustained exercise beyond 4 months potentially leading to even greater improvements (Bernier-Jean et al., 2022). However, Hargrove et al. (2021) suggested that it may take 6 months or more for exercise programmes to maximise the relief of depressive symptoms in this population. Interestingly, Singh et al. (2023) found that the effectiveness of exercise interventions tends to decline with longer durations. Thus, while exercise is beneficial, the specific dose, including type, intensity, frequency and duration, requires further investigation to optimise the improvement of depressive symptoms in HD patients. This study highlights the need for more research into the precise parameters of exercise interventions tailored to this population.

In this systematic review, we integrated data from diverse research on multiple forms of exercise, varying intensities, frequency and duration to elevate the influence of exercise on depressive symptoms in patients with HD. We calculated adherence scores grounded in the ACSM guidelines, categorising subjects into high adherence and low/uncertain adherence groups. Subgroup analyses were subsequently executed to assess the effects of exercise dose on improving depressive symptoms in these individuals.

The subgroup analysis indicated that exercise exhibited a notable beneficial effect on diminishing depressive symptoms in both the high adherence and low/uncertain adherence groups [high adherence SMD = −0.66, 95% CI (−0.91, −0.41); low/uncertain adherence SMD = −0.56, 95% CI (−1.08, −0.05)], with statistical significance (*p* < 0.05). This implies that exercise, regardless of adherence to ACSM guidelines, is beneficial for improving depressive symptoms in HD patients. This aligns with previous studies highlighting the potential of exercise to alleviate depression (Singh et al., 2023). However, our study found that higher adherence to exercise prescriptions was more effective in reducing depressive symptoms than low or uncertain adherence (high SMD = −0.66 > low or uncertain SMD = −0.56).

The meta-analysis underscores the significant benefits of following exercise doses that align closely with ACSM guidelines relative to those with lower or uncertain compliance. These findings have strong clinical relevance and can provide a basis for developing standardised and methodical exercise intervention programmes for HD patients. Therefore, we recommend that healthcare professionals develop tailored exercise plans for HD patients with HD-related depression as early as possible. During exercise therapy, it is crucial to adjust the exercise dose based on individual patient characteristics and gradually increase it to achieve high adherence to ACSM recommendations, while prioritising patient safety. In clinical practice, customising exercise interventions per ACSM recommendations and providing personalised exercise prescriptions for HD patients with depression is essential. Future research should focus on conducting more RCTs that adhere to ACSM guidelines, with larger sample sizes, multicenter involvement and more rigorous study designs. This will further validate our findings and contribute to the development of systematic, standardised and repeatable exercise intervention protocols.

However, this study has several limitations. First, comprehensive outlines of exercise regimens in the interventions are essential for establishing a sensible spectrum of ACSM adherence scores. Unfortunately, the included studies exhibited disparities in the frequency, intensity and duration of exercise, making it difficult to develop common criteria for determining the most effective exercise interventions. Second, some investigations failed to report or inadequately documented exercise intervention doses. Thus, even with strict adherence to ACSM recommendations, exercise doses might be vaguely categorised as belonging to low or uncertain adherence groups. Lastly, the number of qualified RCTs examining the impact of exercise on depression in HD patients remains restricted. This paucity highlights the necessity for additional relevant investigations in this area to enhance the existing knowledge foundation. Overall, while the findings are promising, they warrant careful consideration, given the substantial variability noted across the examined research.

Despite its inherent limitations, this systematic review nonetheless presents valuable practical implications for clinical application. In exploring the optimal exercise dose for depression in HD patients, the exercise dose with high adherence to the ACSM guidelines is associated with better physical health outcomes, a classification that helps improve adherence to tailored exercise prescriptions. The findings support strict adherence to the ACSM recommended dose of exercise as one of the therapeutic strategies to improve depression in HD patients, while emphasizing personalized physical activity prescriptions to maximize the health benefits of exercise.

## 5 Conclusion

In this investigation, we observed that exercise exerts a notable beneficial influence on depressive symptoms in HD patients. Notably, our results suggest that strict adherence to the ACSM’s exercise guidelines has a greater impact on depressive symptoms than non-strict or uncertain adherence. This highlights the potential advantages of adhering to recommended physical activity protocols for HD patients. However, it is essential to recognise that the data derived from the meta-analysis is influenced by the limited number of studies, the unclear extent of participants’ adherence to exercise in specific investigations, and the varying composition of cases across the studies.

## Data Availability

The datasets presented in this study can be found in online repositories. The names of the repository/repositories and accession number(s) can be found in the article/Supplementary Material.
