# Supplementary material for: Effects of exercise dose based on the ACSM recommendations on depression in hemodialysis patients: a systematic review and meta-analysis of randomized controlled trials
Source: Front Physiol. 2025 Jan 31;15:1513746. doi: 10.3389/fphys.2024.1513746 (PMC11825786; doi:10.3389/fphys.2024.1513746)
Supplement: Supplementary file 1 [file Table1.docx]

| Database | Search strategy | amount |
| --- | --- | --- |
|  | PubMed |  |
| #1 | Search: ("Renal Dialysis"[Mesh] OR "Dialysis"[Mesh]) OR "Renal Insufficiency, Chronic"[Mesh] Sort by: Most Recent | [236,694](https://pubmed.ncbi.nlm.nih.gov/?sort=date&term=("Renal+Dialysis"[Mesh]+OR++"Dialysis"[Mesh])+OR+"Renal+Insufficiency,+Chronic"[Mesh]&size=200) |
| #2 | Search: maint* hemodialysis[Title/Abstract] OR H?emodialysis[Title/Abstract] OR dialysis[Title/Abstract] OR renal dialysis[Title/Abstract] OR hemodiafiltration[Title/Abstract] OR Hemodialysis Units, Hospital[Title/Abstract] OR Kidneys, Artificial[Title/Abstract] OR Hemofiltration[Title/Abstract] OR Intermittent Renal Replacement Therapy[Title/Abstract] OR Ultrafiltration[Title/Abstract] OR blood dialysis[Title/Abstract] OR blood purification[Title/Abstract] OR chronic kidney disease[Title/Abstract] OR End-stage renal disease[Title/Abstract] | [240,640](https://pubmed.ncbi.nlm.nih.gov/?term=maint*+hemodialysis[Title/Abstract]+OR+H?emodialysis[Title/Abstract]+OR+dialysis[Title/Abstract]+OR+renal+dialysis[Title/Abstract]+OR+hemodiafiltration[Title/Abstract]+OR+Hemodialysis+Units,+Hospital[Title/Abstract]+OR+Kidneys,+Artificial[Title/Abstract]+OR+Hemofiltration[Title/Abstract]+OR+Intermittent+Renal+Replacement+Therapy[Title/Abstract]+OR+Ultrafiltration[Title/Abstract]+OR+blood+dialysis[Title/Abstract]+OR+blood+purification[Title/Abstract]+OR+chronic+kidney+disease[Title/Abstract]+OR+End-stage+renal+disease[Title/Abstract]&size=200&ac=no&sort=relevance) |
| #3 | #1 OR #2 | [344,174](https://pubmed.ncbi.nlm.nih.gov/?term=(maint*+hemodialysis[Title/Abstract]+OR+H?emodialysis[Title/Abstract]+OR+dialysis[Title/Abstract]+OR+renal+dialysis[Title/Abstract]+OR+hemodiafiltration[Title/Abstract]+OR+Hemodialysis+Units,+Hospital[Title/Abstract]+OR+Kidneys,+Artificial[Title/Abstract]+OR+Hemofiltration[Title/Abstract]+OR+Intermittent+Renal+Replacement+Therapy[Title/Abstract]+OR+Ultrafiltration[Title/Abstract]+OR+blood+dialysis[Title/Abstract]+OR+blood+purification[Title/Abstract]+OR+chronic+kidney+disease[Title/Abstract]+OR+End-stage+renal+disease[Title/Abstract])+OR+(("Renal+Dialysis"[Mesh]+OR+"Dialysis"[Mesh])+OR+"Renal+Insufficiency,+Chronic"[Mesh])&size=200&ac=no&sort=relevance) |
| #4 | Search: "Exercise"[Mesh] Sort by: Most Recent | [260,512](https://pubmed.ncbi.nlm.nih.gov/?sort=date&term="Exercise"[Mesh]&size=200) |
| #5 | Search: Exercise[Title/Abstract] OR activity[Title/Abstract] OR movement[Title/Abstract] OR Resistance Training[Title/Abstract] OR Plyometric Exercise[Title/Abstract] OR Exercise Movement Techniques[Title/Abstract] OR Exercise Therapy[Title/Abstract] OR Endurance Training[Title/Abstract] OR Tai Ji[Title/Abstract] OR Yoga[Title/Abstract] OR Qigong[Title/Abstract] OR Baduanjin[Title/Abstract] OR Yijinjing[Title/Abstract] OR Aerobic Exercises[Title/Abstract] OR Physical Training[Title/Abstract] OR Physical Activity[Title/Abstract] OR physical exercise[Title/Abstract] OR Training[Title/Abstract] OR Motor Activity[Title/Abstract] OR Tai Chi[Title/Abstract] OR Vibration[Title/Abstract] OR wuqinxi[Title/Abstract] OR baduanjin[Title/Abstract] OR yijinjing[Title/Abstract] OR kickboxing[Title/Abstract] OR Pilates[Title/Abstract] OR Balance[Title/Abstract] OR Resistance[Title/Abstract] | [5,308,128](https://pubmed.ncbi.nlm.nih.gov/?term=Exercise[Title/Abstract]+OR+activity[Title/Abstract]+OR+movement[Title/Abstract]+OR+Resistance+Training[Title/Abstract]+OR+Plyometric+Exercise[Title/Abstract]+OR+Exercise+Movement+Techniques[Title/Abstract]+OR+Exercise+Therapy[Title/Abstract]+OR+Endurance+Training[Title/Abstract]+OR+Tai+Ji[Title/Abstract]+OR+Yoga[Title/Abstract]+OR+Qigong[Title/Abstract]+OR+Baduanjin[Title/Abstract]+OR+Yijinjing[Title/Abstract]+OR+Aerobic+Exercises[Title/Abstract]+OR+Physical+Training[Title/Abstract]+OR+Physical+Activity[Title/Abstract]+OR+physical+exercise[Title/Abstract]+OR+Training[Title/Abstract]+OR+Motor+Activity[Title/Abstract]+OR+Tai+Chi[Title/Abstract]+OR+Vibration[Title/Abstract]+OR+wuqinxi[Title/Abstract]+OR+baduanjin[Title/Abstract]+OR+yijinjing[Title/Abstract]+OR+kickboxing[Title/Abstract]+OR+Pilates[Title/Abstract]+OR+Balance[Title/Abstract]+OR+Resistance[Title/Abstract]&size=200&ac=no&sort=relevance) |
| #6 | #4 OR #5 | [5,369,412](https://pubmed.ncbi.nlm.nih.gov/?term=(Exercise[Title/Abstract]+OR+activity[Title/Abstract]+OR+movement[Title/Abstract]+OR+Resistance+Training[Title/Abstract]+OR+Plyometric+Exercise[Title/Abstract]+OR+Exercise+Movement+Techniques[Title/Abstract]+OR+Exercise+Therapy[Title/Abstract]+OR+Endurance+Training[Title/Abstract]+OR+Tai+Ji[Title/Abstract]+OR+Yoga[Title/Abstract]+OR+Qigong[Title/Abstract]+OR+Baduanjin[Title/Abstract]+OR+Yijinjing[Title/Abstract]+OR+Aerobic+Exercises[Title/Abstract]+OR+Physical+Training[Title/Abstract]+OR+Physical+Activity[Title/Abstract]+OR+physical+exercise[Title/Abstract]+OR+Training[Title/Abstract]+OR+Motor+Activity[Title/Abstract]+OR+Tai+Chi[Title/Abstract]+OR+Vibration[Title/Abstract]+OR+wuqinxi[Title/Abstract]+OR+baduanjin[Title/Abstract]+OR+yijinjing[Title/Abstract]+OR+kickboxing[Title/Abstract]+OR+Pilates[Title/Abstract]+OR+Balance[Title/Abstract]+OR+Resistance[Title/Abstract])+OR+("Exercise"[Mesh])&size=200&ac=no&sort=relevance) |
| #7 | Search: (("Mental Disorders"[Mesh]) OR ( "Depressive Disorder"[Mesh] OR "Depression"[Mesh] )) OR "Mood Disorders"[Mesh] Sort by: Most Recent | [1,603,625](https://pubmed.ncbi.nlm.nih.gov/?sort=date&term=(("Mental+Disorders"[Mesh])+OR+(+"Depressive+Disorder"[Mesh]+OR++"Depression"[Mesh]+))+OR+"Mood+Disorders"[Mesh]&size=200) |
| #8 | Search: Mental Disorder[Title/Abstract] OR Mental disorders[Title/Abstract] OR mental symptom[Title/Abstract] OR mental illness[Title/Abstract] OR mental healthor affective disorder[Title/Abstract] OR mental distress[Title/Abstract] OR mental wellbeing[Title/Abstract] OR Depressive Disorder[Title/Abstract] OR Depression[Title/Abstract] OR depressive disorder, major[Title/Abstract] OR major depression[Title/Abstract] OR major depressive disorder[Title/Abstract] OR MDD[Title/Abstract] OR mood disorder[Title/Abstract] OR depress*[Title/Abstract] OR depressed[Title/Abstract] OR distress*[Title/Abstract] OR despair[Title/Abstract] OR fear*[Title/Abstract] OR panic*[Title/Abstract] OR emotion*[Title/Abstract] OR feeling*[Title/Abstract] OR mood[Title/Abstract] OR stress disorders, post traumatic[Title/Abstract] OR posttraumatic stress disorder[Title/Abstract] OR PTSD[Title/Abstract] OR burnout, psychological[Title/Abstract] OR burnout, professional[Title/Abstract] OR burnout[Title/Abstract] OR burn-out[Title/Abstract] | [1,273,154](https://pubmed.ncbi.nlm.nih.gov/?term=Mental+Disorder[Title/Abstract]+OR+Mental+disorders[Title/Abstract]+OR+mental+symptom[Title/Abstract]+OR+mental+illness[Title/Abstract]+OR+mental+healthor+affective+disorder[Title/Abstract]+OR+mental+distress[Title/Abstract]+OR+mental+wellbeing[Title/Abstract]+OR+Depressive+Disorder[Title/Abstract]+OR+Depression[Title/Abstract]+OR+depressive+disorder,+major[Title/Abstract]+OR+major+depression[Title/Abstract]+OR+major+depressive+disorder[Title/Abstract]+OR+MDD[Title/Abstract]+OR+mood+disorder[Title/Abstract]+OR+depress*[Title/Abstract]+OR+depressed[Title/Abstract]+OR+distress*[Title/Abstract]+OR+despair[Title/Abstract]+OR+fear*[Title/Abstract]+OR+panic*[Title/Abstract]+OR+emotion*[Title/Abstract]+OR+feeling*[Title/Abstract]+OR+mood[Title/Abstract]+OR+stress+disorders,+post+traumatic[Title/Abstract]+OR+posttraumatic+stress+disorder[Title/Abstract]+OR+PTSD[Title/Abstract]+OR+burnout,+psychological[Title/Abstract]+OR+burnout,+professional[Title/Abstract]+OR+burnout[Title/Abstract]+OR+burn-out[Title/Abstract]&size=200&ac=no&sort=relevance) |
| #9 | #7 OR #8 | 2,410,833 |
| #10 | #3 AND #6 AND #9 | 2065 |
|  | Embase |  |
| #1 | 'maint* hemodialysis':ab,ti OR h?emodialysis:ab,ti OR dialysis:ab,ti OR 'renal dialysis':ab,ti OR hemodiafiltration:ab,ti OR 'hemodialysis units, hospital':ab,ti OR 'kidneys, artificial':ab,ti OR hemofiltration:ab,ti OR 'intermittent renal replacement therapy':ab,ti OR ultrafiltration:ab,ti OR 'blood dialysis':ab,ti OR 'blood purification':ab,ti OR 'chronic kidney disease':ab,ti OR 'end-stage renal disease':ab,ti | [371,057](https://www.embase.com/) |
| #2 | 'mental disorder':ab,ti OR 'mental disorders':ab,ti OR 'mental symptom':ab,ti OR 'mental illness':ab,ti OR 'mental healthor affective disorder':ab,ti OR 'mental distress':ab,ti OR 'mental wellbeing':ab,ti OR 'depressive disorder':ab,ti OR depression:ab,ti OR 'depressive disorder, major':ab,ti OR 'major depression':ab,ti OR 'major depressive disorder':ab,ti OR mdd:ab,ti OR 'mood disorder':ab,ti OR depress*:ab,ti OR depressed:ab,ti OR distress*:ab,ti OR despair:ab,ti OR fear*:ab,ti OR panic*:ab,ti OR emotion*:ab,ti OR feeling*:ab,ti OR mood:ab,ti OR 'stress disorders, post traumatic':ab,ti OR 'posttraumatic stress disorder':ab,ti OR ptsd:ab,ti OR 'burnout, psychological':ab,ti OR 'burnout, professional':ab,ti OR burnout:ab,ti OR 'burn out':ab,ti | [1,677,355](https://www.embase.com/) |
| #3 | exercise:ab,ti OR activity:ab,ti OR movement:ab,ti OR 'resistance training':ab,ti OR 'plyometric exercise':ab,ti OR 'exercise movement techniques':ab,ti OR 'exercise therapy':ab,ti OR 'endurance training':ab,ti OR 'tai ji':ab,ti OR yoga:ab,ti OR qigong:ab,ti OR 'aerobic exercises':ab,ti OR 'physical training':ab,ti OR 'physical activity':ab,ti OR 'physical exercise':ab,ti OR training:ab,ti OR 'motor activity':ab,ti OR 'tai chi':ab,ti OR vibration:ab,ti OR wuqinxi:ab,ti OR baduanjin:ab,ti OR yijinjing:ab,ti OR kickboxing:ab,ti OR pilates:ab,ti OR balance:ab,ti OR resistance:ab,ti | [6,619,033](https://www.embase.com/) |
| #4 | #1 AND #2 AND #3 | 1,853 |
|  | Web of Science （Web of Science Core Collection） |  |
| #1 | TS=(maint* hemodialysis OR H?emodialysis OR dialysis OR renal dialysis OR hemodiafiltration OR Hemodialysis Units, Hospital OR Kidneys, Artificial OR Hemofiltration OR Intermittent Renal Replacement Therapy OR Ultrafiltration OR blood dialysis OR blood purification OR chronic kidney disease OR End-stage renal disease ) | 163,352 |
| #2 | TS=(Mental Disorder or Mental disorders or mental symptom or mental illness or mental healthor affective disorder or mental distress or mental wellbeing OR Depressive Disorder or Depression or depressive disorder, major or major depression or major depressive disorder or MDD or mood disorder or depress* or depressed OR distress* or despair or fear* or panic* or emotion* or feeling* or mood OR stress disorders, post traumatic or posttraumatic stress disorder or PTSD or burnout, psychological or burnout, professional or burnout or burn-out) | 715,123 |
| #3 | TS=( Exercise OR activity OR movement OR Resistance Training OR Plyometric Exercise OR Exercise Movement Techniques OR Exercise Therapy OR Endurance Training OR Tai Ji OR Yoga OR Qigong OR Baduanjin OR Yijinjing OR Aerobic Exercises OR Physical Training OR Physical Activity OR physical exercise OR Training OR Motor Activity OR Tai Chi OR Vibration OR wuqinxi OR baduanjin OR yijinjing OR kickboxing OR Pilates OR Balance OR Resistance ) | 4,667,644 |
| #4 | #1 AND #2 AND #3 | 1027 |
|  | Cochrane Library |  |
| #1 | (maint* hemodialysis OR H?emodialysis OR dialysis OR renal dialysis OR hemodiafiltration OR Hemodialysis Units, Hospital OR Kidneys, Artificial OR Hemofiltration OR Intermittent Renal Replacement Therapy OR Ultrafiltration OR blood dialysis OR blood purification OR chronic kidney disease OR End-stage renal disease):ti,ab,kw | [43187](http://coch.ilibs.cn/en/advanced-search?searchBy=-1&searchText=maint*+hemodialysis+OR+H?emodialysis+OR+dialysis+OR+renal+dialysis+OR+hemodiafiltration+OR+Hemodialysis+Units,+Hospital++OR+Kidneys,+Artificial+OR+Hemofiltration+OR+Intermittent+Renal+Replacement+Therapy+OR+Ultrafiltration+OR+blood+dialysis+OR+blood+purification+OR+chronic+kidney+disease+OR+End-stage+renal+disease&isWordVariations=&resultPerPage=25&searchType=advanced&forceTypeSelection=true&selectedType=central&displayText=&orderBy=relevancy&p_p_id=scolarissearchresultsportlet_WAR_scolarissearchresults&p_p_lifecycle=0&p_p_state=normal&p_p_mode=view&p_p_col_id=column-1&p_p_col_pos=1&p_p_col_count=2) |
| #2 | (Mental Disorder or Mental disorders or mental symptom or mental illness or mental healthor affective disorder or mental distress or mental wellbeing OR Depressive Disorder or Depression or depressive disorder, major or major depression or major depressive disorder or MDD or mood disorder or depress* or depressed OR distress* or despair or fear* or panic* or emotion* or feeling* or mood OR stress disorders, post traumatic or posttraumatic stress disorder or PTSD or burnout, psychological or burnout, professional or burnout or burn-out ):ti,ab,kw | [224843](http://coch.ilibs.cn/en/advanced-search?searchBy=-1&searchText=Mental+Disorder+or+Mental+disorders+or+mental+symptom+or+mental+illness+or+mental+healthor+affective+disorder+or+mental+distress+or+mental+wellbeing+OR+Depressive+Disorder+or+Depression+or+depressive+disorder,+major+or+major+depression+or+major+depressive+disorder+or+MDD+or+mood+disorder+or+depress*+or+depressed+OR+distress*+or+despair+or+fear*+or+panic*+or+emotion*+or+feeling*+or+mood+OR+stress+disorders,+post+traumatic+or+posttraumatic+stress+disorder+or+PTSD+or+burnout,+psychological+or+burnout,+professional+or+burnout+or+burn-out+&isWordVariations=&resultPerPage=25&searchType=advanced&forceTypeSelection=true&selectedType=central&displayText=&orderBy=relevancy&p_p_id=scolarissearchresultsportlet_WAR_scolarissearchresults&p_p_lifecycle=0&p_p_state=normal&p_p_mode=view&p_p_col_id=column-1&p_p_col_pos=1&p_p_col_count=2) |
| #3 | (Exercise OR activity OR movement OR Resistance Training OR Plyometric Exercise OR Exercise Movement Techniques OR Exercise Therapy OR Endurance Training OR Tai Ji OR Yoga OR Qigong OR Baduanjin OR Yijinjing OR Aerobic Exercises OR Physical Training OR Physical Activity OR physical exercise OR Training OR Motor Activity OR Tai Chi OR Vibration OR wuqinxi OR baduanjin OR yijinjing OR kickboxing OR Pilates OR Balance OR Resistance):ti,ab,kw | [457417](http://coch.ilibs.cn/en/advanced-search?searchBy=-1&searchText=Exercise+OR+activity++OR+movement+OR+Resistance+Training++OR+Plyometric+Exercise+OR+Exercise+Movement+Techniques++OR+Exercise+Therapy++OR+Endurance+Training+OR+Tai+Ji++OR+Yoga+OR+Qigong++OR+Baduanjin++OR+Yijinjing++OR+Aerobic+Exercises+OR+Physical+Training++OR+Physical+Activity+OR+physical+exercise+OR+Training+OR+Motor+Activity+OR+Tai+Chi+OR+Vibration+OR+wuqinxi+OR+baduanjin+OR+yijinjing+OR+kickboxing++OR+Pilates++OR+Balance++OR+Resistance&isWordVariations=&resultPerPage=25&searchType=advanced&forceTypeSelection=true&selectedType=central&displayText=&orderBy=relevancy&p_p_id=scolarissearchresultsportlet_WAR_scolarissearchresults&p_p_lifecycle=0&p_p_state=normal&p_p_mode=view&p_p_col_id=column-1&p_p_col_pos=1&p_p_col_count=2) |
| #4 | #1 AND #2 AND #3 | 1132 |
| Database | Search strategy | amount |
